# Dendritic turnover zones support switching between rules without expanding the cortical engram

**DOI:** 10.64898/2026.08.26.747194

**Published:** 2026-10-16

**Authors:** Ioanna Pandi, Spyridon Chavlis, Hatem Oraby, Mostafa A. Nashaat, Matthew E. Larkum, Athanasia Papoutsi, Panayiota Poirazi

## Abstract

Flexible behavior depends on frontal circuits that use experience to adjust subsequent decisions. Yet how synaptic connections in these circuits are structurally reorganized when new associations compete with established ones remains unclear. Here, we used a novel rule-switching behavioral paradigm, longitudinal spine imaging, and circuit modeling to investigate how dendrites accommodate changing associations. Inactivation of secondary motor cortex (M2) impaired rule switching but not learning or maintenance in control conditions. Switching increased spine turnover on apical tuft dendrites in M2, concentrating spine formation, elimination, and new-spine clustering within persistent dendritic turnover zones, with pre-existing spines lost early during adaptation. The model reproduced these dynamics and predicted preferential elimination of synapses encoding the prior rule. Preventing dendritic compartment reuse increased the neuronal population representing the rules. These findings suggest that persistent turnover zones support synaptic reorganization during behavioral updating, with reuse offering a potential economy in neuronal recruitment.

## Full Text Availability

The license terms selected by the author(s) for this preprint version do not permit archiving in PMC. The full text is available from the preprint server.

